# Current Targeted Therapy Options in the Treatment of Cholangiocarcinoma: A Literature Review

**DOI:** 10.7759/cureus.26233

**Published:** 2022-06-23

**Authors:** Ekaterina Proskuriakova, Anwar Khedr

**Affiliations:** 1 Internal Medicine, Mount Sinai Hospital, Chicago, USA; 2 Internal Medicine, Tanta University Faculty of Medicine, Tanta, EGY; 3 Internal Medicine, BronxCare Health System, New York, USA

**Keywords:** biliary cancer, bile duct cancer, treatment of cholangiocarcinoma, molecularly targeted therapy, intrahepatic cholangiocarcinoma, advanced biliary tract cancer

## Abstract

Biliary cancer (BC) is a rare disease. It is formed from the biliary epithelium of the small ducts in the liver periphery (intrahepatic) and the main ducts of the hilum (extrahepatic). The incidence of intrahepatic carcinoma is rising in the western world, and the incidence of gallbladder cancer is declining. Surgical treatment is the primary treatment option for localized forms of these tumors, but only a small group of patients is eligible for it. Palliative therapy is the standard treatment option for those with advanced cases, and it mainly relies on chemotherapy. The advanced form's five-year survival of BC does not exceed 5%. However, targeted therapy aimed at tumors with fusion mutations of the fibroblast growth factor receptor (FGFR), isocitrate dehydrogenase (IDH) 1 and 2, the genes encoding the B-Raf protein (*BRAF*), breast cancer (*BRCA1/2*), epidermal growth factor receptor 2 (*HER2*), and the neurotrophic receptor tyrosine kinase gene (*NTRK*), is gradually changing the paradigm in the treatment of this disease. This can be seen especially in approaches to treating intrahepatic cholangiocarcinoma, which is associated with a high incidence of mutations. This literature review aims to provide the latest scientific evidence regarding targeted therapy in treating cholangiocarcinoma.

## Introduction and background

Biliary cancer (BC) accounts for 3% of the total number of gastrointestinal tumors [1]. The disease is more common among men than women (the number of cases is 1.7 and 1.3 per 100 thousand people per year, respectively) [2]. BC ranks second among all tumors of the hepatobiliary system and is anatomically subdivided into intrahepatic cholangiocarcinoma, distal cholangiocarcinoma, hepatic hilum cancer, and gallbladder tumors [3]. BC, which is formed from the epithelium of the small biliary tracts in the peripheral part of the liver, is called intrahepatic, and those that are formed from the ducts in the hilum of the liver are extrahepatic. The last one includes gallbladder cancer, ampullary cancer, and the cancer of the ducts in the pancreas [4]. Even though in the western world, the incidence of BC is not that high, around 0.35 to 2 per 100,000 per year; in China and Thailand, it will be up to 40-times higher [5,6]. According to recent data, the incidence of intrahepatic cholangiocarcinoma is rising. The data from the USA and other countries have shown a persistent and stable rising incidence from 0.1 cases per 100,000 to 0.6 per 100,000 during the period of the last 30 years [5,6]. Regarding gallbladder cancer, the incidence was shown to be comparatively uniform or decreasing in high-income countries, mainly due to the increase in routine cholecystectomy [7]. Approximately 220,000 new cases were reported worldwide in 2018, with the highest incidence found in women from the southern part of Chile (27 cases per 100,000) [8].

The difference in incidence between countries reflects the discrepancy in various risk factors in these places. The main risk factors of the cholangiocarcinoma are considered to be primary sclerosing cholangitis, hepatolithiasis, liver fluke infections, cirrhosis, hepatitis B and C, fatty liver disease, and diabetes [9]. However, it is essential to mention that most patients with cholangiocarcinoma may have no known underlying risk factors. While in the western world, cholangiocarcinoma is associated mainly with chronic inflammation, in Thailand, liver fluke (*Opisthorchis viverrini)* is the most significant risk factor, which is provoked by eating raw or undercooked fish for over 20 years. In addition, *Chlonorhis sinesis* is the primary organism that causes this cancer in people in China, Korea, and Taiwan [10]. 

**Figure 1:**
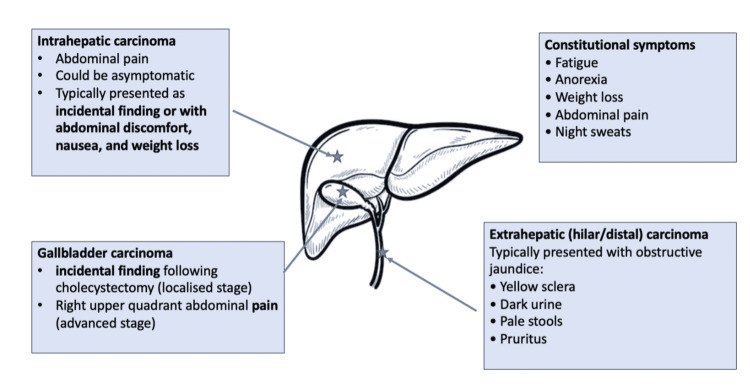
Clinical presentation of the BC

The incidence of gallbladder cancer increases with age and is more spread among women. The main reason for this type of cancer, which is accountable for 70-90% of gallbladder cancer, is a history of choledocholithiasis. Nevertheless, only around 3% of gallstones will lead to the development of cancer [11]. The symptoms of the BC vary between the anatomical location of the primary tumor and are summarized in Figure 1 [4]. 

Diagnosis of the cholangiocarcinoma should be started with a thorough investigation of the risk factors, along with the liver function tests and blood tests, exploring signs of infections and biliary tract obstruction, including raised white blood cell count, blood cultures, neutrophilia, and elevation of the C-reactive protein. Ultrasound examination could be used as the primary imaging exam for the identification of the obstruction or dilatation of the biliary tree, following CT as the primary method for the diagnosis and staging of cancer [12]. MRI with the hepato-specific contrast media and diffusion-weighted imaging could provide information about vascular involvement and a more detailed anatomical presentation of the lesion [13]. 18F-fluorodeoxyglucose positron emission tomography can be used in addition to CT and MRI to identify lymph node involvement, distal metastasis development, and recurrent disease after surgery. This technique is usually used before planning a surgery [14]. Serum carbohydrate antigen 19-9 is not specific to the cholangiocarcinoma and could be raised in other diseases, but if it's present in a patient with confirmed BC, it could inform about the response to treatment and prognosis [15]. Endoscopic retrograde cholangiopancreatography (ERCP), with a specificity of 70%, provides an opportunity to acquire brush cytology and biopsy [16], while endoscopic ultrasound is used to assess and take a sample of the distal cholangiocarcinoma and regional lymph nodes [17]. 

Gallbladder cancer is found predominantly in women, and extrahepatic cholangiocarcinoma and intrahepatic cholangiocarcinoma are more common among men [18]. Due to the fact that currently, the five-year survival rate for the advanced process in cholangiocarcinoma does not exceed 5% [19], a detailed study of BC is required in order to improve the results in a group of patients with this diagnosis. 

Surgical treatment is the primary treatment option for localized forms of these tumors, with a recurrence rate of approximately 55% [20]. Liver resection established on the anatomical involvement is performed for the intrahepatic cholangiocarcinomas, while a pancreatoduodenectomy may be required for the extrahepatic cholangiocarcinoma. Gallbladder carcinomas are mainly incidentally found during non-oncological procedures. A repeat resection may be necessary [4]. However, the relapse rate remains high and varies from 42% to 70%, according to the latest data [21]. Therefore, since 2017 adjuvant six-month chemotherapy with capecitabine, which has become the standard of care based on the latest randomized phase III study, should be offered to patients after successful resection of cholangiocarcinoma or gallbladder cancer [22-24]. 

According to the BILCAP study, adjuvant treatment with capecitabine versus placebo showed no significant statistical improvement relative to the primary endpoint of overall survival (OS) among all randomized patients. However, a survival study adjusted for other prognostic factors, such as lymph node status, grade of malignancy, and gender, showed a positive effect on mean overall survival (53 months in the capecitabine group versus 36 months in the placebo group) [21].

Only a small group of patients is eligible for surgical treatment, so palliative therapy is the standard treatment option for those with advanced cases. The five-year survival rate for this group of patients with non-resectable tumors is approximately 5-10%. The first-line therapy based on the clinical study ABC-02 for such patients is cisplatin/gemcitabine (CisGem) [25]. 

In phase III clinical study ABC-02 conducted in 2019, 410 patients with non-resectable BC were randomized into two treatment groups (CisGem and gemcitabine) [25]. The superiority of the CisGem group in terms of OS (11.2 months) compared to the second group (8.1 months), p < 0.001, was established. Patients who received combination had also improved progression-free survival (PFS) (8.0 months vs 5.0 months, p<0.001) and tumor control rate, 81.4% in CisGem and 71.8% in gemcitabine group, p=0,049 [25]. Similar data were obtained in a Japanese phase II clinical study BT22. Median OS 11.2 months was shown to be in combination group and 7.7 months in gemcitabine group (HR 0.69, 95% CI 0,42-1,13) [26].

The standard second-line treatment after CisGem is the fluorouracil plus oxaliplatin (FOLFOX) regimen, based on the 2019 Phase III Study ABC-06. During this study, 81 patients were randomized either to the FOLFOX group or the symptomatic control group. A slight superiority of the FOLFOX group in terms of median overall survival (6.2 months) compared to the other group of patients (5.3 months) was found (p = 0.031). At the same time, the overall response rate was statistically insignificant [27].

**Figure 2:**
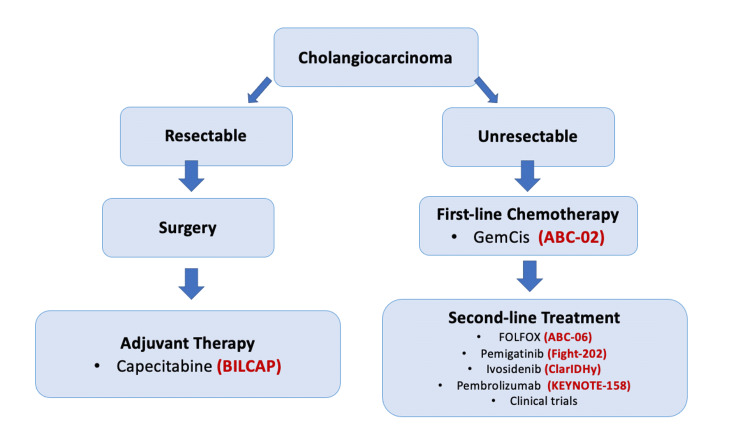
Treatment strategy in cholangiocarcinoma. GemCis, gemcitabine + cisplatin; FOLFOX, 5-fluorouracil + oxaliplatin Copyright/License: Licensee: Cancer Science, Tokyo, Japan. This figure is from an open-access article distributed under the terms and conditions of the Creative Commons Attribution (CC BY) license. (http://creativecommons.org/licenses/by/4.0/). Modifications were made to the original figure. Zhang R, Puzzoni M, Mariani S, et al.: Emerging treatment evolutions and integrated molecular characteristics of biliary tract cancers. Cancer Sci. 2021, 112:4819-4833. https://doi.org/10.1111/cas.15139 [29].

At the moment, the palliative treatment of BC mainly relies on chemotherapy [28]. Figure 2 shows the treatment strategy in patients with cholangiocarcinoma [29].

One of the most innovative cancer treatment methods today is targeted therapy which contributes to changing approaches to cancer care. Targeted therapy allows attacking specific genes or proteins that play a role in the development and survival of the tumor cells. This therapy is the treatment approach commonly seen in the management of intrahepatic cholangiocarcinoma, which is associated with a high incidence of fibroblast growth factor receptor (*FGFR*) fusion mutations and dehydrogenase isocitrate (*IDH*)-1 and 2 mutations [4]. In addition, there are other potent targets in BC, such as the B-Raf protein-encoding gene (*BRAF*), breast cancer genes (*BRCA1/2*), epidermal growth factor receptor 2 (*HER2*), anaplastic lymphoma tyrosine kinase receptor (*ALK*), receptor tyrosine kinase-encoding proto-oncogene (*RET*), and neurotrophic receptor tyrosine kinase gene (*NTRK*), which are actively being investigated [4]. 

Modern treatment of BC with targeted therapy has been carefully studied and will be summarized in this review to highlight the current treatment options for patients with cholangiocarcinoma. 

## Review

Role of FGFR in the treatment of cholangiocarcinoma 

BC is associated with a diverse list of mutations. The most responsive to targeted therapy driver mutation groups have been determined and summarized in Table 1 [4].

**Table 1:** Therapeutic targets and incidence of mutations in biliary tract cancers. FGFR- fibroblast growth factor receptor; IDH -1 -  dehydrogenase isocitrate 1 mutations; *BRAF* - B-Raf protein-encoding gene; *NTRK* - neurotrophic receptor tyrosine kinase gene; *ERBB2* (*HER2*) -  epidermal growth factor receptor 2

Mutation/Pathway	Targeted agents	Incidence of mutation in BC
FGRF pathway	Infigratinib Erdafitinib Pemigatinib Derazantinib	FGFR1-3 Fusion: Intrahepatic Cholangiocarcinoma – 11 - 12.5%; Extrahepatic Cholangiocarcinoma – 0 % ; Gallbladder Cancer - 3%
IDH1	Ivosidenib	IDH1/2 Substitution: Intrahepatic Cholangiocarcinoma – 15-23%; Extrahepatic Cholangiocarcinoma – 3-4 % ; Gallbladder Cancer - 0%
BRAF	Dabrafenib plus trametinib	BRAF Substitution: Intrahepatic Cholangiocarcinoma – 5% ; Extrahepatic Cholangiocarcinoma – 3 %; Gallbladder Cancer - 1%
NTRK	Entrectinib Larotrectinib	Rare
ERBB2 (HER2)	Pertuzumab and trastuzumab Neratinib	ERRB2 amplification: Intrahepatic Cholangiocarcinoma – 3% ; Extrahepatic Cholangiocarcinoma – 11% ; Gallbladder Cancer - 16%

FGFRs are a family of four transmembrane receptors with an intracellular tyrosine kinase domain (FGFR 1-4) [1]. When the FGFR receptor is activated, the Ras/RAF/MEK, JAK/STAT, and PI3K/Akt pathways are subsequently activated [2]. FGF signaling disruption is associated with proliferation, malignant cell migration, and angiogenesis in a large number of tumors [19]. 

The most frequent change in the FGFR receptor in BC is the fusion of genes, occurring in 10-16% of patients with intrahepatic cholangiocarcinoma. Point mutations and gene amplification are also observed, but these changes are rarely diagnosed in this group of patients [3]. FGFR mutations are uncommon in patients with extrahepatic cholangiocarcinoma and gallbladder cancer [3]. The first fusion gene detected in 2013 was *FGFR2-BICC1*, and scientists subsequently identified more than 50 other chimeric genes [18]. 

Studies of specific and selective FGFR inhibitors such as infigratinib, derazantinib, erdafitinib, pemigatinib, futibatinib, and debio 1347 are currently underway [22]. Most of these drugs can reversibly bind to cysteine residues on the P-loop in the pocket of adenosine triphosphate acid (ATP). Selective FGFR inhibitors showed significant clinical activity in patients with advanced cholangiocarcinoma refractory to treatment if patients had acquired the fusion FGFR gene. To date, two drugs have been approved: pemigatinib (approved by the United States Food and Drug Administration (FDA) in 2020 and the European Medicines Agency (EMA) in 2021) and infigratinib (approved by the FDA in 2021) [23]. 

The final results of the phase II infigratinib study were presented in 2021 at a symposium on gastrointestinal cancer organized by the American Association of Oncologists (ASCO) [24]. A multicenter study of 108 patients with the identified fusion *FGFR2* gene treated for cholangiocarcinoma in the later stages of the disease found that infigratinib showed an overall response rate of 23.1% with a mean response time (RT) of 5 months and a mean progression-free survival (mPFS) of 7.3 months [24]. The most frequent side effects were hyperphosphataemia, fatigue, stomatitis, and baldness, with grade 3-4 side effects being hyponatremia and hypo- or hyperphosphataemia [24].

The next treatment for patients with cholangiocarcinoma targeting the FGFR receptor is derazantinib [30]. In addition to blocking the FGFR receptor, this drug also inhibits other kinases, such as RET, VEGFR1, DDR, and KIT [31]. The role of derazantinib was evaluated in the open-label phase I/II clinical trials of Mazzaferro et al. This study enrolled 29 patients with intrahepatic cholangiocarcinoma and the fusion FGFR gene who did not receive first-line chemotherapy or received but did not have a successful outcome. Derazantinib (300 mg daily) showed an overall survival rate of 20.7% and a tumor control rate of 82.8% [32]. 

Erdafitinib is an inhibitor of all four FGFR receptors [28]. Despite unsatisfactory results in the treatment of urothelial carcinoma, there is not much data on the efficacy of erdafitinib as a treatment for patients with cholangiocarcinoma and FGFR mutations. Phase IIa clinical trial results were presented in early 2022. The study enrolled 232 patients from Asian countries with cholangiocarcinoma. Twenty-two patients received erdafitinib 8 mg once daily, every 28 days. The OS rate was 40.9%, and PFS was 5.6 months [27]. 

Pemigatinib is the first approved drug for treating severe cholangiocarcinoma as an inhibitor of FGFR1, FGFR2, and FGFR3 receptors [33]. In FIGHT-202, a phase II, multicenter, open-label clinical trial, 146 patients were divided into three groups: patients with fusion FGFR receptor (N=107), patients with other FGFR receptor changes (N =20), and patients without receptor changes (N=18). With a mean follow-up of 17.8 months, 38 patients (35.5%) from the first and second groups achieved an objective response (three patients with complete response). The mean duration of response was 7.5 months. The duration of response ≥ 6 months was found in 68% of patients and ≥ 12 months in 37% of patients. Based on the results of this study, in April 2020, pemigatinib received FDA approval for the treatment of cholangiocarcinoma with metastasis, with FGFR mutations identified by FoundationOne CDx [34]. This test is a type of qualitative next-generation sequencing that uses targeted hybridization-based capture technology to detect substitutions, insertions, and delete alterations in 324 genes. It is used to provide tumor mutation profiling for patients with solid malignant neoplasms [34].

Despite the fact that this group of drugs is actively used for the treatment of patients with cholangiocarcinoma, there are a number of questions that are not answered. For example, questions about the presence of resistance to this targeted therapy at an early stage or subsequent stages of treatment remain unclear [35]. Primary and secondary resistance is an important topic in the study of treatment of patients with cholangiocarcinoma. Currently, the main mechanisms of resistance development are known, but there is little information on how to overcome it. Additionally, hyperphosphatemia, onycholysis, alopecia, mucositis, conjunctivitis, other visual disorders, myalgia, and joint pain are the main (specific) side effects of this group of drugs. Non-specific side effects are fatigue, anorexia, diarrhea, and hepatotoxicity [1]. Table 2 shows the clinical outcome of selective FGFR inhibitors in treating patients with cholangiocarcinoma [23]. 

**Table 2:** Selective FGFR inhibitors: Clinical development in treating patients with cholangiocarcinoma ORR- Overall response rate, PFS - progression-free survival

Drug	Infigratinib [9], (n= 71)	Erdafitinib [14], (n=232)	Derazantinib [12], (n=20)	Pemigatinib [16], (n=107)
ORR	23.1%	40.9 %	20.7%	35.5%
PFS	7.3 months	5.6 months	5.7 months	7.5 months

Role of IDH 1/2 mutation in the treatment of patients with cholangiocarcinoma 

IDH 1/2 is an enzyme involved in regulating DNA metabolism and repair [36]. Typically, this enzyme catalyzes the conversion of isocitrate to alpha-ketoglutarate. However, mutations in this gene can lead to epigenetic changes like an increase in the production of 2-hydroxyglutarate, an oncometabolite, which in turn can cause disruptions in DNA and the methylation of histones [37]. The concentration of this oncometabolite can be measured in serum and can potentially become a biomarker based on the fact that in patients with IDH 1/2 mutations, the concentration of the oncometabolite was significantly increased compared to patients without mutations [38]. Mutations in the IDH 1/2 gene occur in approximately 20-25% of patients with intrahepatic cholangiocarcinoma and are almost undetectable in patients with other localized BC [39].

For patients with advanced cholangiocarcinoma refractory to one or more lines of treatment with a mutation in the IDH 1/2 gene, ivosidenib, an inhibitor of this gene, in phase III clinical trial ClarIDHy, showed efficacy compared to placebo. Patients were randomized 2:1 to ivosidenib and placebo. Patients were allowed to crossover, i.e., switch from placebo to ivosidenib at disease progression [40]. The study showed improvement in mPFS in the placebo group (1.4 months) and the ivosidenib group (2.7 months), with a p < 0.001. Absolute mean progression-free survival may not appear clinically significant; however, it should be noted that progression-free survival rates at six months (32.0%) and 12 months (21.9%) were better in the ivozidenib arm. The median OS in the ivozidenib group was 10.3 months, and in the placebo group, only 7.5 months (p = 0.09). Based on the data obtained, ivosidenib was approved by the FDA as a new standard of care for patients with intrahepatic cholangiocarcinoma with an IDH 1/2 mutation refractory to chemotherapy [41]. The main side effects in the treatment with ivosidenib were fatigue (26%), nausea (36%), diarrhea (31%), and ascites being the most common grade 3 side effect (7%) [40]. Table 3 shows the results of the ClarIDHy trial [40]. 

**Table 3:** Ivosidenib in patients with IDH1-mutant, chemotherapy-refractory cholangiocarcinoma (ClarIDHy) PFS - progression-free survival, mOS- median overall survival

	Ivosidenib [22] (n=121)	Placebo (n=59)
PFS	2.7 months	1.4 months
PFS at 6 months	32.0 %	-
PFS at 12 months	21.9%	-
mOS	10.3 months	7.5 months

The role of *BRAF*/*MEK* mutations in the treatment of cholangiocarcinoma

**Figure 3:**
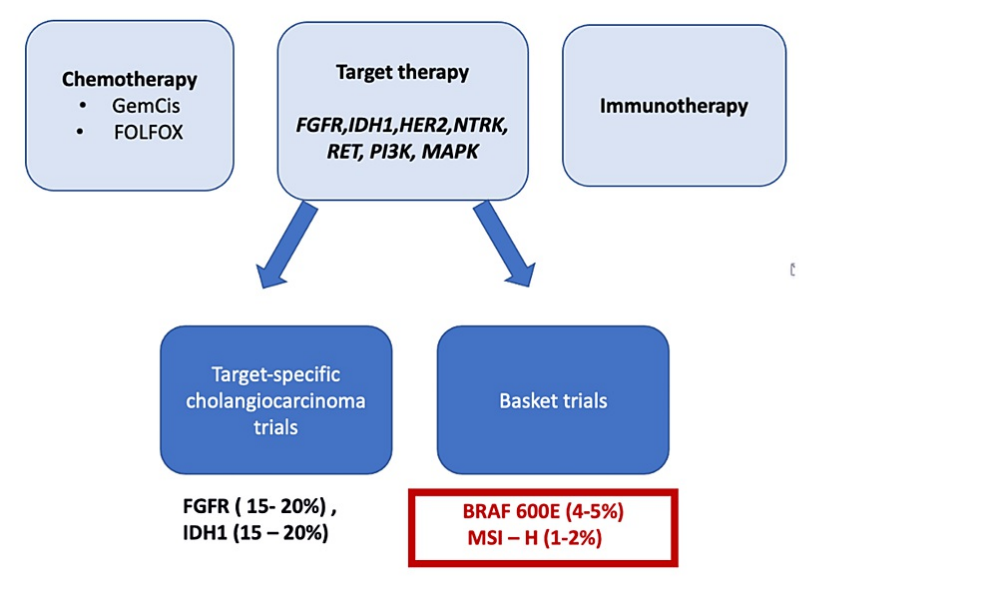
Systemic treatment for cholangiocarcinoma FGFR - fibroblast growth factor receptor, IDH1 - isocitrate dehydrogenase 1, HER2 - epidermal growth factor receptor 2, NTRK - the neurotrophic receptor tyrosine kinase gene, PI3K - phosphatidylinositol 3-kinase, RET - receptor tyrosine kinase-encoding proto-oncogene, MAPK - mitogen-activated protein kinase, BRAF - the genes encoding the B-Raf protein, MSI-H - high levels of microsatellite instability

Mitogen-activated protein kinase/extracellular signal-regulated kinase, or MEK signaling pathway, is involved in cell proliferation and survival and is a frequent mutation in tumor development. The most potent activator of this pathway is the mutation in the *BRAF* gene. The most frequent mutation in this gene is the activating mutation resulting from the replacement of glutamate with valine (V600E) [42]. Despite the frequent occurrence of melanoma and papillary thyroid cancer, the frequency of this mutation in patients with BC is low (1-6%), mainly with intrahepatic localization [43]. Treatment options for patients with cholangiocarcinoma are summarized in Figure 3 [23].

A phase II clinical trial of ROAR in 178 patients with BRAF V600E mutation, including 33 patients with advanced and refractory BC, evaluated the efficacy of the combination of dabrafenib, a BRAF inhibitor, and trametinib, a MEK inhibitor, in the treatment of various types of tumors [44]. With a mean follow-up of 8 months, the overall response rate was 41%, the mean progression-free survival was 7.2 months, and the mean overall survival was 11.3 months. It has been established that this may be an effective combination in this small group of patients [45]. 

*NTRK* inhibitors 

The *NTRK* gene encodes the Trk receptor, a member of the tyrosine kinase family that is associated with the MAPK signaling pathway upon activation [44]. The chimeric NTRK gene may be a target for treatment strategies for some cancers. This chimeric gene results in activation of the Trk receptor, which stimulates cancer cells' differentiation, proliferation, and survival. The chimeric NTRK gene was detected in 3.5% of patients with intrahepatic cholangiocarcinoma. Trk inhibitors may also be effective against ROS1 and alk mutations, with incidence rates in patients with cholangiocarcinoma of 8.7% and 2.7 %, respectively [46]. 

Trk receptor inhibitors that are important in the treatment of BC at this stage are entrectinib [47] and Larotrectinib [48]. Using a tumor-independent approach, i.e., a study-targeted mutation type rather than a tumor localization or type, an impressive overall survival rate (57-75%) was shown with a complete response rate of 7-16% in the group of patients with solid masses who had failed pretreatment. These products have been approved by the FDA and EMA [47,48]. 

HER2 amplification 

The HER family includes four receptors: Epidermal Growth Factor Receptor (EGFR/HER1), HER2, HER3, and HER4 [49]. About 5-15% of BCs have a mutation in HER2 and are most commonly expressed in gallbladder cancer and extrahepatic cholangiocarcinoma [50]. Potential working targets of this molecular pathway are changes in the *HER2* gene itself (known as *ERBB2*) [51], amplification, and overexpression of *HER2*. Anti-HER2 therapy with targeted agents, pertuzumab and trastuzumab, showed significant improvement in outcomes in patients with other types of cancer, such as breast cancer and gastrointestinal tumors (gastric cancer and gastroesophageal cancer [51]. 

A phase IIa, non-randomized, multicenter MyPathway trial evaluated the activity of targeted therapy with pertuzumab in combination with trastuzumab in patients with various types of solid tumors (basket trial) with acquired, potentially active *HER2* genetic mutation. In a group of 32 patients with biliary cancer refractory to chemotherapy who experienced amplification, overexpression, or both in the *HER2* gene, the overall survival rate was 23% [52].

Neratinib (an irreversible inhibitor of HER1, HER2, and HER4) showed an overall survival rate of 12%, with a decrease in gallbladder tumor size or extrahepatic location in patients with advanced BC and *HER2* mutation [53]. Meanwhile, the median overall survival was 5.4 months, and progression-free survival was 2.8 months [53]. 

Limitations

While we searched for material for this review, there were some limiting factors. Our data were primarily obtained from the free access articles only in the English language; therefore, some articles of closed access and written in other languages may have been missed. This article highlighted the main molecular targets, but not all known mutations, in treating patients with cholangiocarcinoma and did not discuss the conservative treatment, including surgical management in detail. 

## Conclusions

Treatment of BC is a rapidly developing area of oncology, with a large number of clinical studies that show impressive results. The management of a patient with an advanced, unresectable cholangiocarcinoma today still mainly relies on highly toxic chemotherapy, the basis of which is CisGem as the first line of therapy and the FOLFOX regime as a second line. However, the tumor genetic profiling done by next-generation sequencing (NGS) provides an opportunity to detect mutations (targets) and classify them into separate molecular subsets. This has changed the paradigm in treating this cancer as there is no shortage of those targets, and more than 40% of patients with this BC have them. If there are targeted mutations, the patient has a chance to delay the cumulative chemotherapy-related toxicity and has a better prognosis for survival.

One of the most promising therapies is targeted FGFR alterations, and more than 40 inhibitors targeting this pathway are in clinical development. However, the main challenges of these groups of drugs continue to be resistance which will be an area of many studies over the following years. Regarding IDH 1 inhibitors, which are found in around 20% of patients with BC, the area of future interest is the development of the covalent inhibitor of IDH1 that allows increasing potency in IDH1 mutations. Basket trials, which allow studying mutations in various tumors, have also boosted the research in BC. Patients with a BC diagnosis could be involved in studies with well-known therapies for other types of cancer and have tumor shrinkage in cholangiocarcinoma. DNA damage repair (DDR), MDM2 oncoprotein, and p53 studies could also benefit the patients with cholangiocarcinoma, and all are under investigation nowadays.

Currently, identification of the molecular groups with the known molecular therapies is a rapidly emerging area. Therefore, it is essential for a clinician to look for those mutations and to plan subsequent treatment adequately. Due to the large selection of treatment methods for advanced BC, achieving maximum results in the treatment of BC and increasing the life expectancy of many patients is possible.
